# Pedobarographic and kinematic analysis in the functional evaluation of two post-operative forefoot offloading shoes

**DOI:** 10.1186/s13047-015-0116-3

**Published:** 2015-10-29

**Authors:** Paolo Caravaggi, Alessia Giangrande, Lisa Berti, Giada Lullini, Alberto Leardini

**Affiliations:** Movement Analysis Laboratory and Functional-Clinical Evaluation of Prostheses, Istituto Ortopedico Rizzoli, Via di Barbiano 1/10, 40136 Bologna, Italy

**Keywords:** Offloading shoes, Kinematics, Kinetics, Plantar pressure, Lower limb joints

## Abstract

**Background:**

Forefoot offloading shoes are special orthopaedic footwear designed to protect and unload the injured part of the foot after surgery and for conservative treatments.

The offloading action is often achieved by transferring plantar load to the rearfoot via rocker shoes with reduced contact area between shoe and ground. While these shoes are intended to be worn only for short periods, a compromise must be found between functionality and the risk of alterations in gait patterns at the lower limb joints. In this study, the pedobarographic, kinematic and kinetic effects of a traditional half-shoe and a double-rocker full-outsole shoe were compared to those of a comfortable shoe (control).

**Methods:**

Ten healthy female participants (28.2 ± 10.0 years) were asked to walk in three different footwear conditions for the left/right foot: control/half-shoe, control/full-outsole, and control/control. Full gait analysis was obtained in three walking trials for each participant in each condition. Simultaneously a sensor insole system recorded plantar pressure in different foot regions. Normalized root-mean-square error, coefficient of determination, and frame-by-frame statistical analysis were used to assess differences in time-histories of kinematic and kinetic parameters between shoes.

**Results:**

The half -shoe group showed the slowest walking speed and the shortest stride length. Forefoot plantar load was significantly reduced in the half-shoe (maximum force as % of Body Weight: half-shoe = 62.1; full-outsole = 86.9; control = 93.5; *p* < 0.001). At the rearfoot, mean pressure was the highest in the full-outsole shoe. At the ankle, sagittal-plane kinematics in the full-outsole shoe had a pattern more similar to control.

**Conclusions:**

The half-shoe appears significantly more effective in reducing plantar load at the forefoot than a double-rocker full-outsole shoe, which is designed to reduce forefoot loading by using an insole with a thicker profile anteriorly as to maintain the foot in slight dorsiflexion. However, the half-shoe is also associated with altered gait spatio-temporal parameters, more kinematic modifications at the proximal lower limb joints and reduced propulsion in late stance.

**Electronic supplementary material:**

The online version of this article (doi:10.1186/s13047-015-0116-3) contains supplementary material, which is available to authorized users.

## Background

The use of specific orthopaedic shoes is often recommended for foot conservative treatments and after surgery. Patients are required to wear forefoot offloading shoes (FOS) following several types of forefoot interventions, such as the surgical correction of hallux valgus and of lesser toe deformities, and in the treatment of plantar ulcers in the diabetic foot [1–3]. FOS are intended to protect and unload the injured part of the foot via a large variety of designs [4–6]. One of the most traditional and widely-used FOS design, known as half-shoe - “talus-shoe” and “reverse camber” are other less common definitions [7] - features a low-profile outsole in the forefoot region. The half-shoe has been shown to be highly effective in reducing forefoot pressure in gait; however, due to its peculiar design, this FOS is liable to discomfort and instability [8–12].

While several shoe designs for forefoot offloading have been proposed to address these issues, only few have been assessed thoroughly by established functional analysis before being introduced in the market. A FOS featuring a short-outsole design has proved to be extremely effective in reducing forefoot pressure when compared to the half-shoe, in either appropriate and inappropriate use – i.e. while attempting to put weight on the forefoot [8]. However the participants recruited in the study reported a more comfortable walk when wearing the latter shoe. In a further development, a modification of the short-outsole design has proved to increase its comfort though at the expense of the total forefoot offloading [11]. Four different FOS designs were compared with a cast and a control shoe in 24 neuropathic diabetic patients at high risk of plantar ulceration [10]. Reduction of forefoot pressure was observed in all shoes but, according to the VAS score, walking comfort was the lowest in the half-shoe. Stability, comfort and rolling characteristics were analyzed, though only qualitatively, together with pressure measures, in 11 different types of post-operative shoes obtained with several combinations of external shapes and internal materials [13]. Inconsistent results were observed, thus leading to the general conclusion that the selection of the most appropriate shoe type depends on specific foot conditions and subjective wearing characteristics.

Ideally, FOS should perform their offloading action with minimum kinematic and kinetic alterations at the lower limbs which could generate instability during walking in both the affected and unaffected leg. In the half-shoe, due to the peculiar shape of the outsole, the alteration in lower limb joints kinematics is expected to be particularly significant, especially in the third rocker of stance. Although a comprehensive understanding of the functional outcome of modern FOS requires the integration of several measurement systems, only few studies have considered additional non-pedobarographic measures [13, 14]. It has been shown that wearing elevated orthopaedic shoes causes higher loads in the lower limb joints and significantly increases hip adduction and pelvic tilt on the ipsilateral side, unless it is compensated by an equivalent heel height on the contralateral foot [15]. More recently, stability and risk of falls in the half-shoe have been investigated by analysing the modifications in body dynamics and foot pressure [16].

Therefore, the purpose of this study was to use pedobarography, alongside state-of-the-art gait analysis, to compare the effects of a full-outsole shoe on kinematics and kinetics of lower limb joints with those in a traditional half-shoe, while a normal comfortable shoe was used as a control. The present thorough functional assessment aims at achieving a better understanding of the effectiveness of these FOS designs to obtain forefoot offloading whilst preserving normal joint kinematics and kinetics. In order for the results not to be affected by different clinical histories and treatments, the study was conducted on a population of healthy participants.

## Methods

### Participants

Ten young female participants (age 28.2 ± 10.0 years; height 1.64 ± 0.04 m; weight 55.1 ± 3.7 kg; BMI 20.4 ± 1.2 kg/m^2^; 38 ± 1 shoe Euro-size), without any lower limb pathology or history of trauma or surgery, volunteered in the study. Female only participants were chosen because they represent the large majority of patients undergoing forefoot surgery (e.g. hallux-valgus) at the authors’ Institute. Approval was granted from the Scientific Committee of Istituto Ortopedico Rizzoli (July 26, 2010, board resolution n° 362)*.* Informed consent was obtained from all participants prior to their participation in the study.

### The tested shoes

**Fig. 1:**
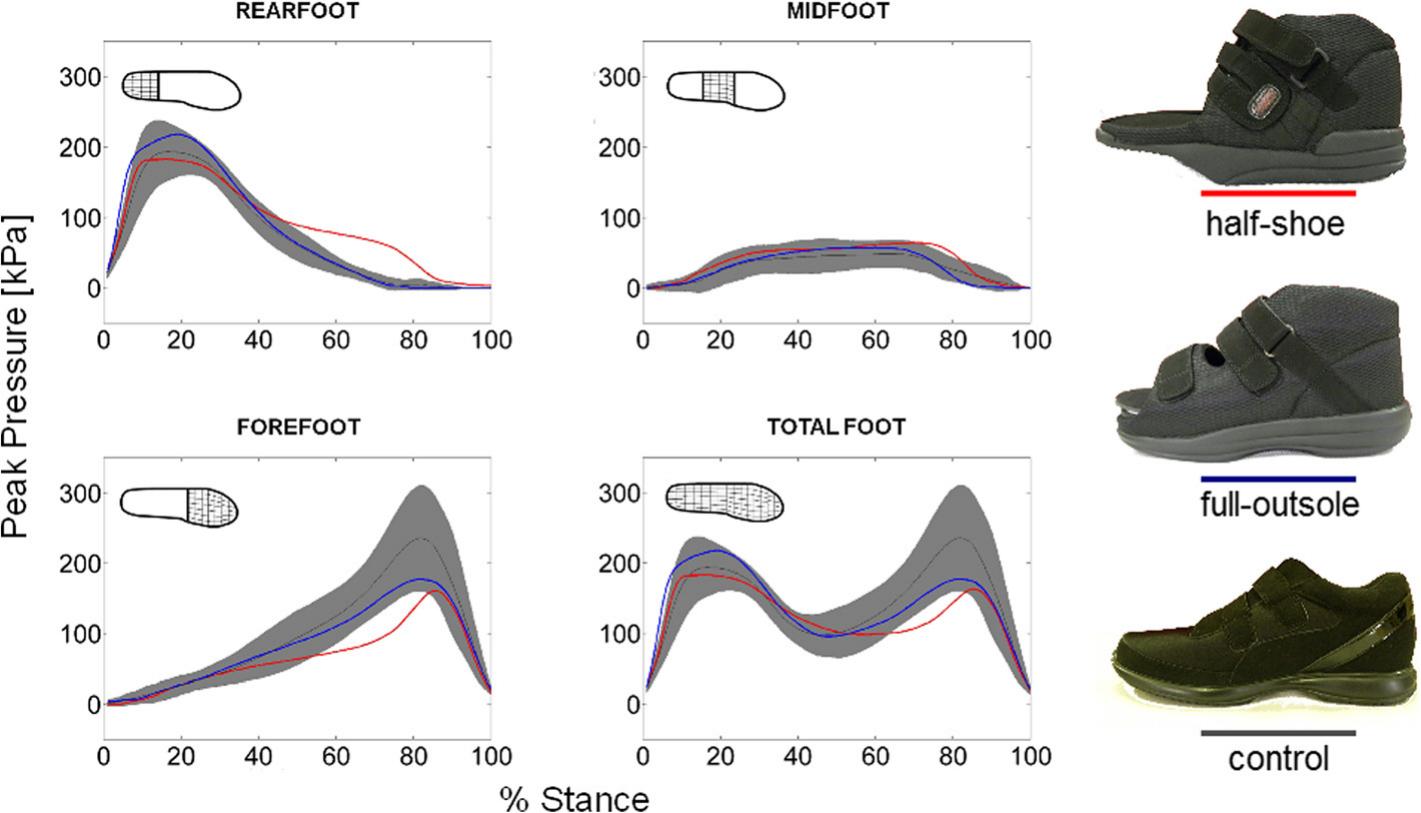
Region-based temporal profiles of peak pressure. *Left*, time-histories of peak pressure (kPa) for each shoe condition at rearfoot, midfoot, forefoot and total foot. Temporal patterns are shown as mean values and standard deviation bands. Right, the three shoes tested in the study (*top to bottom*): a traditional half-shoe, the full-outsole shoe and the control shoe

Two post-operative FOS (Fig. 1, right) were used in the study: a traditional half-shoe (base to tip height difference = 18 mm, pivot point = 55 % of shoe length), and a full-outsole shoe (base to tip height difference = 18 mm) presenting a rigid double-rocker outsole and an insole with an increasingly thicker profile from the rearfoot to the forefoot (2 to 12 mm). Both designs were compared to a standard comfortable shoe assumed as control (Fig. 1, bottom right). The three shoes are produced by the same company (Podartis, Treviso, Italy). Each participant was required to walk at self-selected normal walking speed wearing the control shoe on the left foot and one of the three shoes on the right foot: half-shoe, full-outsole shoe, and control shoe. This aimed at replicating a real clinical scenario in which FOS are worn only on the affected side. The order of the tested shoes was randomised for each participant.

### Pedobarographic measurements

Pressure was measured under both feet and for each footwear condition during walking using the Pedar sensor insoles (Novel GmbH, Munich, Germany) at 100 Hz. Each insole consists of 99 capacitive sensors (pressure range 15-600 kPa; resolution 2.5-5 kPa). This was placed on the shoe internal insole, and was connected to a Bluetooth transmission unit. The repeatability of the system in measuring standard pedobarographic parameters in different foot regions has been extensively demonstrated [17, 18]. Several walking trials were recorded for each participant to allow familiarization with each shoe design and the measurement apparatus. Three steps from each participant were recorded and included in the analysis, on both ipsilateral and contralateral sides in each shoe configuration, for a total of 30 samples in each shoe group across the ten participants. These were recorded, together with the ground reaction forces and lower limb kinematics (see next paragraph), in the central cycles of three five-step walking trials. Analysis of the in-shoe pressure distribution was performed with ad-hoc scripts in Matlab® (The MathWorks, Inc.) by dividing each sensor insole into several regions of interest [8, 10]: rearfoot (0–30 % insole length); midfoot (31–60 % insole length); forefoot (61–100 % insole length). Within the latter, the first metatarsal and the hallux regions were identified. The regions were defined a-priori by selecting the relevant pressure sensors in the insoles. The following pedobarographic parameters were determined in each of these five regions, and for the total insole, over the stance phase of the gait cycle: mean and peak pressure (measured in kPa); mean and maximum force (expressed as percentage of body weight, %BW); pressure–time (PTI, kPa*s) and force-time (FTI, %BW*s) integrals.

### Kinematic and kinetic measurements

Kinematics and kinetics of the pelvis and the main lower limb joints were obtained via an eight-camera stereophotogrammetric system (Vicon Motion Capture, Oxford, UK) sampling at 100 Hz, and ground reaction force via two dynamometric platforms (Kistler Instrument, Winterthur, Switzerland) sampling at 2000 Hz. Joint rotations (in degrees) and moments (in %BW * height) were calculated from the trajectories of 22 reflective markers positioned on relevant lower limb anatomical landmarks according to the IORgait marker set [19, 20]. Note that the three markers tracking the foot – i.e. those on the posterior calcaneus and on the head of first and fifth metatarsals – could not be attached directly to the participants’ skin in the full-outsole and control shoes, thus these were attached instead to appropriate positions on the shoe upper. Range of motion (ROM) and maximum/minimum sagittal-plane rotations of the main lower limb joints [21] were calculated for both ipsilateral (i.e. where the two FOS were worn) and contralateral (i.e. where the control shoe was worn) sides. Walking speed was measured by tracking the position of one marker on the rearfoot.

### Statistical analysis

Non-parametric Friedman’s test was used to assess for statistical differences in the corresponding pedobarographic, kinematic and kinetic parameters between the three footwear conditions. Individual differences between shoes were established via Tukey-Kramer post-hoc test (*α* = 0.05). The median values of pedobarographic parameters have been reported in the text as numerical data.

Comparison of the joints kinematic and kinetic temporal profiles in the two FOS, with the corresponding patterns in the control shoe, was performed in three ways: a) using the coefficient of determination (R-squared), as quantitative score for the shape-similarity between shoe configurations – where R^2^ = 1 indicates temporal profiles perfectly correlated, and R^2^ = 0 profiles not correlated -; b) calculating the normalized root-mean-square error (%NRMSE), as percentage of the total range recorded in the control shoe group, representing the average across stride duration of the frame-by-frame offset between mean temporal profiles, and c) via frame-by-frame non-parametric statistical comparison using Mann–Whitney *U* test [22].

## Results

### Spatio-temporal parameters and ground reaction force

The half-shoe showed walking speed lower than control (m/s, half-shoe = 1.07 ± 0.17; full-outsole = 1.18 ± 0.17; control = 1.24 ± 0.19; *p* < 0.001) and the shortest stride length (in meters, half-shoe = 1.28 ± 0.08; full-outsole = 1.36 ± 0.07; control = 1.39 ± 0.09; *p* < 0.001).

In late stance, both FOS showed decreased antero-posterior component of the ground reaction force (%BW, half-shoe = 17 ± 4 (*p* = 0.014); full-outsole = 17 ± 4 (*p* = 0.0091); control = 22 ± 4), whereas the half-shoe showed also a lower peak of the vertical component (%BW, half-shoe = 107 ± 8; control = 116 ± 7; *p* = 0.0018).

### Pedobarography

Most of the pedobarographic parameters in the half-shoe were significantly different from the control at all five regions, whereas in the full-outsole shoe these were mainly different from those in the half-shoe (Additional file 1: Table S1a).

#### Mean and peak pressure

**Fig. 2:**
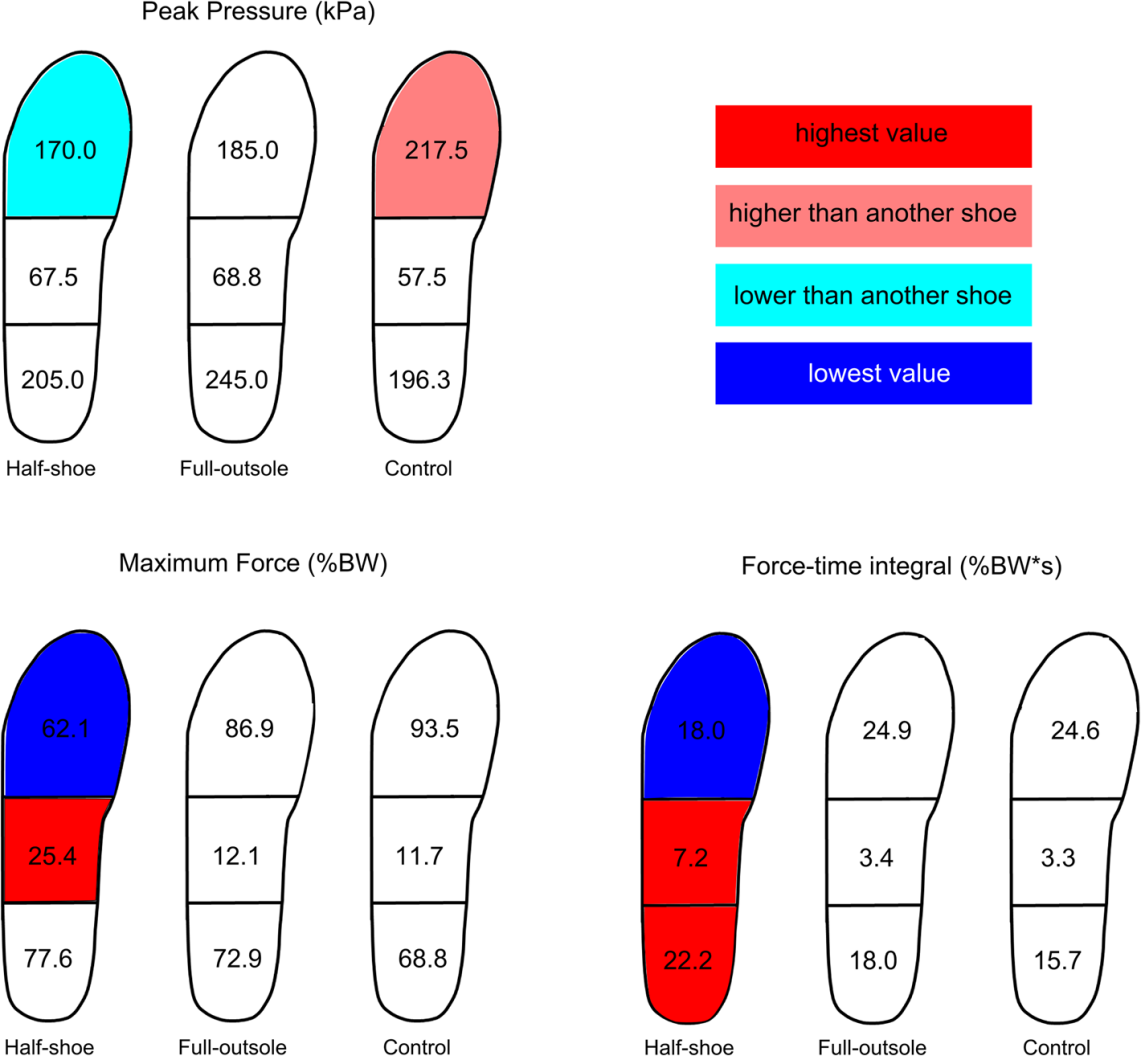
Pedobarography: statistical outcome of comparisons between shoe conditions. Graphical representation of the statistically significant differences (*p* < 0.05) in the main pedobarographic parameters between shoe conditions in the rearfoot, midfoot and forefoot regions of the right foot. The median of each parameter across 30 samples has been shown within the corresponding foot region. See Additional file 1: Table S1a for further details

Both FOS showed differences in peak pressure profiles with respect to control, although the full-outsole shoe showed values more similar to the control (Fig. 1, Additional file 2: Figure S1). In the half-shoe, mean and peak pressure were lower than control at forefoot (Fig. 2). The full-outsole shoe showed the largest mean pressure in the rearfoot (kPa, half-shoe = 102.9 (*p* = 0.021); full-outsole = 120.3 (*p* = 0.044); control = 101.1).

#### Mean and maximum force

In the half-shoe, maximum force was the largest at midfoot and the lowest at forefoot (Fig. 2). Mean force was larger than control at midfoot and the lowest at forefoot (Additional file 1: Table S1a).

#### Force-time and pressure–time integrals

In the half-shoe, FTI was the largest at rearfoot and midfoot and the lowest at forefoot (Fig. 2). PTI was larger than control at midfoot and lower than control at the forefoot (kPa*s, half-shoe = 52.8; control = 63.2; *p* = 0.009). In the full-outsole, no difference was detected in FTI and PTI with respect to control in any foot region.

#### Contralateral limb

In the contralateral limb – i.e. where the control shoe was worn - no significant differences in the pedobarographic parameters were observed between the three shoe groups (Additional file 1: Table S1b), with the exception of FTI in the total foot which was larger in the half-shoe group compared to the control (%BW*s, half-shoe = 49.7; control = 47.3; *p* = 0.009).

### Kinematics

**Fig. 3:**
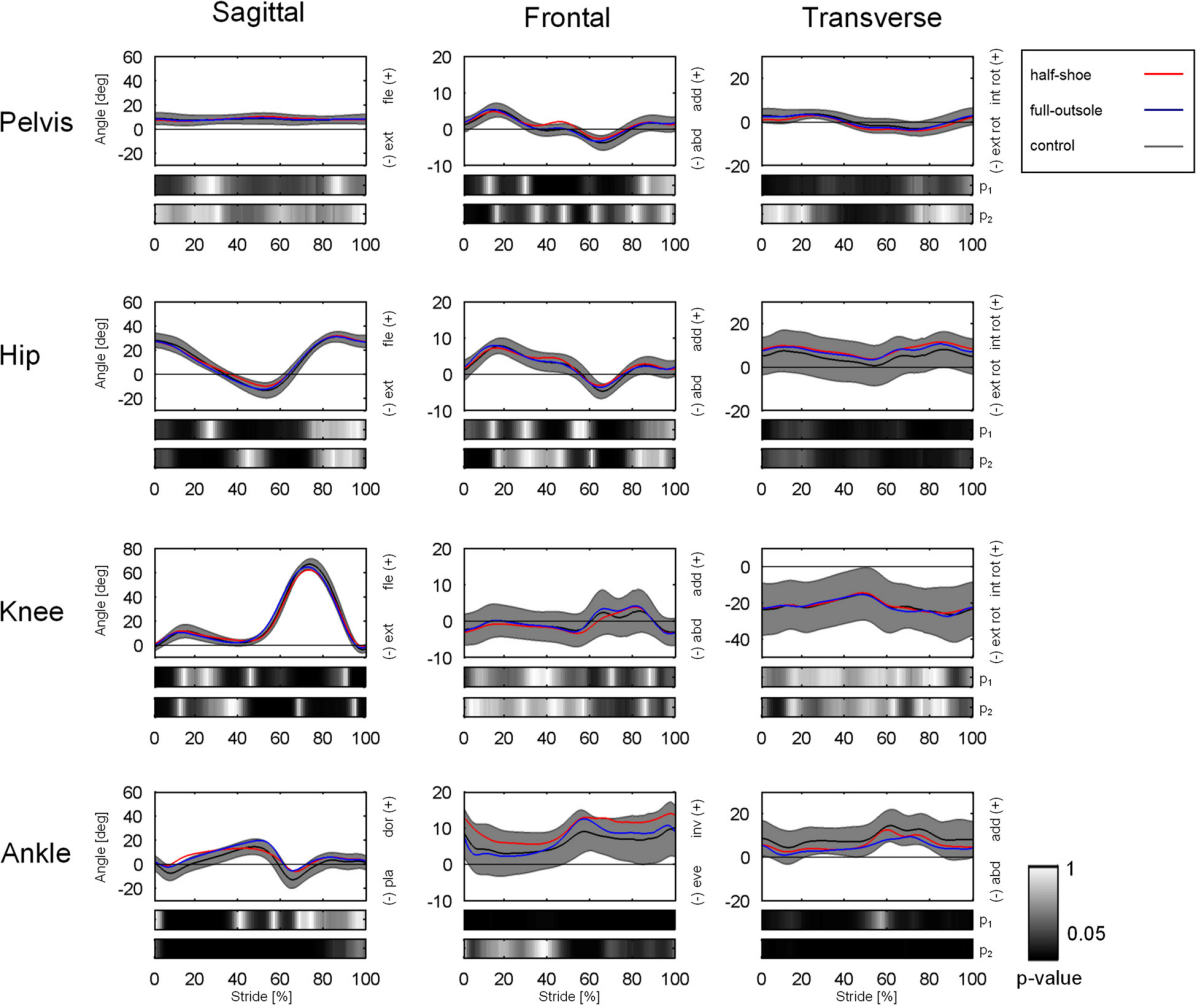
Temporal profiles of joint rotations. Top to bottom, rotations (deg) at the pelvis, hip, knee and ankle joints in the sagittal, frontal and transverse planes (*left to right*) over normalized stride duration. Temporal patterns for each shoe condition are shown as mean values and standard deviation bands. Frame-by-frame color-maps of the statistical p-values between half-shoe and control (p1), and between full-outsole shoe and control (p2), are shown at the bottom of each plot

In general, mean patterns of three – dimensional rotations in the lower limb joints in both FOS designs were strongly correlated to the corresponding patterns in the control (Fig. 3, Table 2).

#### Pelvis

Analysis of the similarity coefficients showed that motion of the pelvis in the full-outsole shoe group was more similar to the control (Fig. 3, Table 2). The sagittal-plane motion of the pelvis in the half-shoe was the most altered across all joints and anatomical planes with respect to the control (R^2^ = 0.27; NRMSE = 68.1 %).

#### Hip

The half-shoe showed a smaller maximum extension in stance at the hip compared to the control (Table 1). Relatively large NRMSE were detected in both FOS but in the transverse plane only (Table 2).

**Table 1:** Kinematic parameters in the ipsilateral limb. Range of motion and maximum/minimum sagittal-plane rotation angles [deg] at the hip, knee and ankle joint in the right side for the three shoe conditions. Kinematic parameters were determined according to [18]

		Half-Shoe	Full-Outsole	Control	*p*	*p*	*p*
		[deg]	[deg]	[deg]	HS vs FO	HS vs CON	FO vs CON
HIP	ROM	44.0	46.4	46.7	0.321	0.158	0.917
	sagittal-plane	(39.4 46.3)	(41.0 48.3)	(43.0 48.5)			
	ROM	12.1	12.0	12.5	0.981	0.866	0.943
	frontal-plane	(11.4 13.3)	(10.9 13.1)	(11.3 14.3)			
	ROM	9.9	10.2	10.6	0.575	0.097	0.535
	transverse-plane	(7.2 13.5)	(7.4 14.5)	(7.2 13.8)			
	Max flexion in swing	30.3	30.7	32.0	0.886	0.962	0.744
		(27.8 35.9)	(28.0 36.0)	(28.4 35.4)			
	Max extension in stance	−10.7*	−11.4	−13.2	0.166	0.028	0.734
		(−15.7 −0.9)	( −16.4 −9.5)	(−17.1 −9.9)			
KNEE	ROM	67.3	68.5	71.4	0.917	0.028	0.077
	sagittal-plane	(60.4 72.2)*	(64.2 72.4)	(69.2 74.2)			
	ROM	9.9	11.1	10.8	0.715	0.682	0.998
	frontal-plane	(7.1 12.6)	(6.5 13.1)	(8.5 13.7)			
	ROM	14.9	16.6	15.6	0.747	0.784	0.998
	transverse-plane	(11.2 17.7)	(13.1 19.1)	(12.2 18.4)			
	Max flexion at loading response	12.4	13.2	14.2	0.999	0.973	0.983
		(9.4 15.7)	(5.5 15.5)	(10.2 15.5)			
	Max extension in stance	2.3	2.1	1.1	0.978	0.978	0.915
		(−0.3 3.6)	(−1.0 4.6)	(0.2 3.0)			
ANKLE	ROM	25.1	27.2	28.1	0.308	0.287	0.999
	sagittal-plane	(21.6 27.5)	(24.5 30.1)	(26.0 30.3)			
	ROM	11.1	11.4	9.4	0.929	0.321	0.529
	frontal-plane	(9.7 12.9)	(8.2 14.4)	(7.3 13.0)			
	ROM	12.7	11.6	13.0	0.692	1.00	0.695
	transverse-plane	(10.5 17.6)	(9.6 14.1)	(10.7 15.0)			
	Max dorsiflexion	13.6	20.1	16.7	0.111	0.971	0.065
	in stance	(6.8 23.7)	(12.9 29.0)	(10.6 19.6)			
	Max plantarflexion at loading response	−0.9	−4.2	−7.0	0.911	0.046	0.015
		(−10.7 4.6)*	(−9.3 6.1)*	(−10.6 −2.2)			

*denotes statistically significant difference between any FOS and control (*p* < 0.05)

**Table 2:** Shape-similarity and offset scores of kinematic and kinetic temporal profiles. Coefficient of determination (R-squared) and normalized root-mean-square error (%NRMSE) of comparisons of kinematic (Fig. 3) and kinetic (Fig. 4) temporal patterns between each FOS and control

Rotations		Sagittal		Frontal		Transverse	
		R^2^	%NRMSE	R^2^	%NRMSE	R^2^	%NRMSE
Pelvis	half-shoe	0.27	68.1	0.93	10.6	0.97	19.0
	full-outsole	0.67	26.7	0.97	5.3	0.97	8.5
Hip	half-shoe	0.99	4.8	0.97	6.3	0.93	44.6
	full-outsole	0.99	3.6	0.97	5.1	0.95	34.7
Knee	half-shoe	1.00	3.4	0.87	15.2	0.96	6.2
	full-outsole	0.99	3.6	0.96	13.0	0.90	9.1
Ankle	half-shoe	0.85	17.4	0.94	57.9	0.93	28.8
	full-outsole	0.93	17.9	0.89	25.7	0.91	41.6
Moments							
Hip	half-shoe	0.95	6.9	0.96	6.3	0.97	11.6
	full-outsole	0.96	5.6	0.92	8.7	0.92	8.1
Knee	half-shoe	0.83	11.8	0.94	7.9	0.89	15.9
	full-outsole	0.87	8.7	0.91	9.0	0.88	12.4
Ankle	half-shoe	0.95	16.3	0.92	16.3	0.95	7.8
	full-outsole	0.99	3.7	0.98	12.8	0.99	9.2

#### Knee

A significantly smaller sagittal-plane ROM was observed at the knee in the half-shoe (Table 1).

#### Ankle

The sagittal-plane rotation pattern of the ankle joint in the full-outsole shoe group was more similar to control (Fig. 3, Table 2). However, similarly to the half-shoe, the full-outsole shoe showed a significant offset in dorsiflexion (Fig. 3) and the largest, albeit not statistically significant (*p* = 0.065), maximum dorsiflexion (Table 1). This was also reflected in reduced maximum plantar-flexion at load response (Table 1). An inversion offset was also detected in the half-shoe throughout the gait cycle.

#### Contralateral limb

In the contralateral limb – i.e. the left side, where only the control shoe was worn - no significant differences in the kinematic parameters were detected between the three shoe conditions on the right side (Additional file 3: Table S2).

### Kinetics

**Fig. 4:**
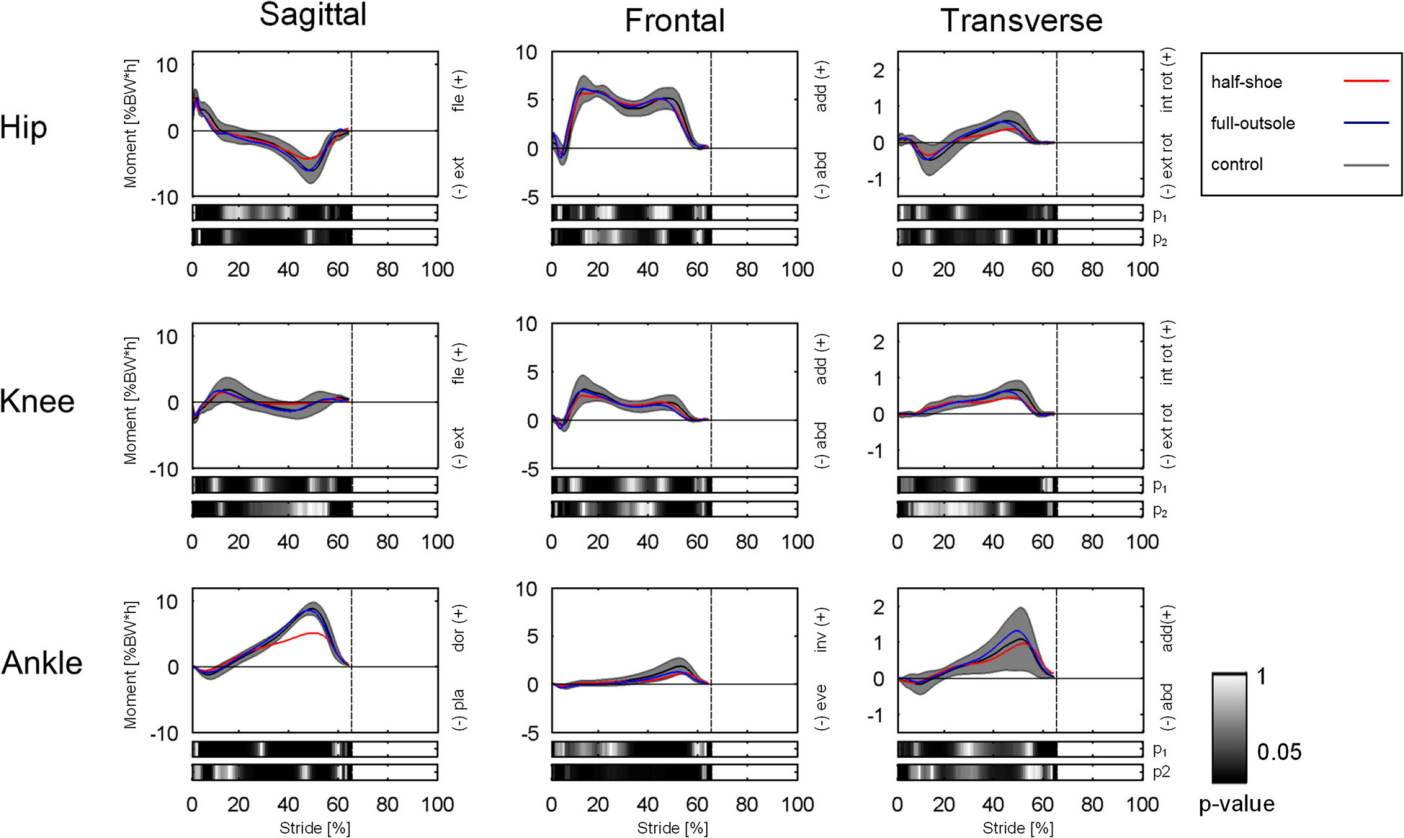
Temporal profiles of joint moments. Top to bottom, moments (%BW*h) at the hip, knee and ankle joints in the sagittal, frontal and transverse planes (*left to right*) over normalized stride duration. Temporal patterns for each shoe condition are shown as mean values and standard deviation bands. Frame-by-frame color-maps of the statistical p-values between half-shoe and control (p1), and between full-outsole shoe and control (p2), are shown at the bottom of each plot

Mean profiles of three – dimensional external moments at lower limb joints in both FOS designs were strongly correlated to the corresponding patterns in the control (Fig. 4, Table 2). Difference in NRMSE between the two FOS was lower than 4 % across all joints and anatomical planes, with exception of the sagittal-plane moment at the ankle joint where the moment was much higher in the half-shoe group.

The half-shoe showed the smallest peak ankle flexion moment in late stance (%BW*h, half-shoe = 5.3 ± 1.7; full-outsole = 8.7 ± 1.0; control = 9.1 ± 0.8; *p* = 0.0010).

## Discussion

The use of FOS is recommended after surgery to unload the anterior part of the foot, in case of injuries and ulcers, and for conservative treatments. While the effectiveness of the half-shoe in reducing forefoot pressure has been demonstrated extensively, concerns have been raised over the peculiar design of the outsole, which features a high heel and a low-profile elevated outsole in the forefoot region, thus potentially causing unstable and inefficient gait dynamics and affecting wearing comfort [16]. These factors have become increasingly important in the design of orthopaedic footwear, and are particularly critical when dealing with elderly patients with pre-existing balance problems, hence at higher risk of fall. In order to find a good compromise between gait performance and forefoot plantar pressure relief, original shoe designs have recently entered into the market [13, 15]. In fact, a randomized single-blind study has shown how a conventional running shoe can help relieve forefoot pressure without compromising the comfort [23]. Nevertheless, the half-shoe is still the most investigated FOS in the literature, even though its evaluation is usually limited to the analysis of pressure distribution via sensor insoles. Very little relevance has thus far been given to the effects of this FOS on the lower limb kinematic chain.

In this study, we aimed at comparing the effectiveness of a full-outsole double-rocker shoe with that of a traditional half-shoe in achieving forefoot offloading whilst preserving normal lower limb joints function. The participants were instructed to walk at their comfortable walking speed in each shoe condition. Wearing the half-shoe proved to be more challenging to the participants and resulted in slightly lower walking speed, shorter stride length and reduced ground reaction force in late stance. While the peculiar shape of the shoe outsole is likely to be the main responsible for the reduction in forefoot pressure and alteration of foot and lower limb kinematics, the effect of the reduced walking speed on these parameters should not be disregarded [22, 24–26].

In fact, similarly to what has been reported in previous studies [8–12], the half-shoe was effective in reducing plantar load at forefoot. Load appears to be transferred mainly to the midfoot, as revealed by the increased force and FTI in this region. In the full-outsole shoe design, forefoot offloading is meant to be achieved by using an insole with an increasingly thicker profile from the rearfoot to the forefoot as also to maintain the ankle slightly in dorsiflexion (Table 1, Fig. 3). The present study shows that this effectively helped shifting foot loading towards the rearfoot, where mean pressure and mean force were found to be higher than the control. At the forefoot however, while peak pressure was not different to that observed in the half-shoe, no pedobarographic parameters were significantly lower than the corresponding values in the control shoe. Most of the pedobarographic parameters were found significantly different in the two FOS at all regions. In the rearfoot mean pressure was the largest in the full-outsole shoe, whereas at midfoot maximum force and FTI were the largest in the half-shoe. According to the model proposed by Jacob [27], internal forefoot joint loading during gait is more affected by the external forces under the hallux compared to those under the metatarsal bones. The half-shoe resulted in significantly smaller force and pressure under the hallux and first metatarsal compared to the control, whereas the full-outsole shoe resulted in some offloading only at the first metatarsal – albeit not statistically different from control. Consequently, it is plausible for the half-shoe to be also more effective in reducing metatarso-phalangeal joints loading.

The kinematic analysis revealed that the full-outsole shoe design results in less alteration at the more proximal lower limb joints The error in sagittal-plane pelvis motion (68.1 %, see Table 2) was the largest across all joints and anatomical planes when wearing the half-shoe, and the participants were performing shorter strides which may have accounted for the reduced hip maximum extension and knee ROM. In addition, while the full-outsole shoe was associated to larger ankle dorsiflexion, the half-shoe presented a larger inversion with respect to the control shoe. These kinematic alterations could exacerbate pre-existing balance problems in some cohorts, therefore the use of the half-shoe might not be the optimal solution to allow early mobilization of these patients. In relation to moments at the more proximal joints, no major differences were detected between the two FOS with respect to the control values. As expected, due to the peculiar low-profile of the anterior part of the outsole, the half-shoe was associated to significantly lower propulsion as observed in the reduced antero-posterior ground reaction force and ankle moment in the sagittal plane at push-off.

Although FOS are usually worn for a limited period of time, discomfort, postural and gait instability, and leg asymmetry have the potential to cause dysfunction to the lower extremity and the back. In the present study, almost no significant alterations in the pedobarographic parameters and in lower limb joints kinematics were detected in any shoe group on the contralateral side. This can be explained by the small leg length discrepancy between FOS and control shoe (lower than 4 mm), but also a likely-consequence of the good musculo-skeletal control achieved by the young and healthy participants recruited in this study.

The results of this study should be critically considered in relation to some limitations. Due to the limited sample size, and in order to achieve statistical power of 0.80, no Bonferroni correction could be applied to account for the multiple comparisons in the kinematic and pedobarographic parameters. Nonetheless, the probability of Type I errors seems extremely low, since the present results were statistically confirmed by several parameters. In order to avoid interference with the shoes, the reflective marker tracking the position of the lateral malleolus was attached in a little more proximal location. However, this position was maintained across all shoe configurations to avoid possible bias in measuring ankle motion. Moreover, in the full-outsole and in the control shoes, the three markers tracking the foot were attached to the corresponding positions on the shoe upper. Standard markers attachment to the participants’ skin could instead be achieved in the half-shoe. While the authors do not believe this inconsistent implementation of the markers’ attachment to be accountable for the different kinematics recorded between the three shoe configurations, some attention should be paid when interpreting the kinematic data. Furthermore, additional studies should be performed before generalizing the present results, initially taken from a sample of young and healthy females, to older subjects or patients undergoing forefoot surgery, as additional biomechanical and clinical factors should be introduced in the analysis.

## Conclusions

The traditional half-shoe appears significantly more effective in reducing plantar load at the forefoot than a double-rocker full-outsole shoe, which is designed to reduce forefoot loading by using an insole with a thicker profile anteriorly. However, the half-shoe is also associated with altered gait spatio-temporal parameters, more kinematic modifications at the proximal lower limb joints and reduced propulsion in late stance.

While the gait parameters recorded in the full-outsole shoe are encouraging further development, additional studies have to be sought to confirm whether the amount of pressure reduction achievable with a full-outsole design is clinically suitable also for post-operative patient cohorts.

## Additional files

Additional file 1: Tables S1.
**a**Pedobarographic parameters in the ipsilateral side. Main pedobarographic parameters in different foot regions and in the total foot in the right side. Median (25 % 75 %) values were calculated across 30 samples for each shoe condition.*denotes statistically significant difference (p<0.05) between any FOS and control. § denotes statistically significant difference (p<0.05) between the two FOS. **b** Pedobarographic parameters in the contralateral side.Main pedobarographic parameters at different foot regions and in the total foot in the left side (where the control shoe was worn). Median (25 % 75 %) values were calculated across 30 samples for each of the three shoe conditions on the right side. *denotes statistically significant difference (p<0.05) between any FOS and control. (ZIP 41 kb) Additional file 2: Figure S1. Sensor-based peak pressure. For each shoe condition, color-map of the median of the peak pressure (kPa) for each sensor across all steps and all participants. (PNG 66 kb) Additional file 3: Table S2. Kinematic parameters in the contralateral limb.Range of motion and maximum/minimum sagittal-plane rotation angles [deg] at the hip, knee and ankle joint in the left side (where the control shoe was worn), for each of the three shoe conditions on the right side. Kinematic parameters were determined according to [18]. (DOCX 14 kb)
