# Supplementary material for: Pedobarographic and kinematic analysis in the functional evaluation of two post-operative forefoot offloading shoes
Source: J Foot Ankle Res. 2015 Oct 29;8:59. doi: 10.1186/s13047-015-0116-3 (PMC4625618; doi:10.1186/s13047-015-0116-3)
Supplement: Additional file 1: Tables S1. — aPedobarographic parameters in the ipsilateral side. Main pedobarographic parameters in different foot regions and in the total foot in the right side. Median (25 % 75 %) values were calculated across 30 samples for each shoe condition.*denotes statistically significant difference (p<0.05) between any FOS and control. § denotes statistically significant difference (p<0.05) between the two FOS. b Pedobarographic parameters in the contralateral side.Main pedobarographic parameters at different foot regions and in the total foot in the left side (where the control shoe was worn). Median (25 % 75 %) values were calculated across 30 samples for each of the three shoe conditions on the right side. *denotes statistically significant difference (p<0.05) between any FOS and control. (ZIP 41 kb) [file 13047_2015_116_MOESM1_ESM.zip › Additional file 1/1514672762170943_add3.docx]

|  |  | **Mean pressure**  **[kPa]** | **p** | **Peak pressure**  **[kPa]** | **p** | **PTI**  **[kPa*s]** | **p** | **Mean force**  **[% BW]** | **p** | **Maximum force**  **[%BW]** | **p** | **FTI**  **[% BW*s]** | **p** |
| --- | --- | --- | --- | --- | --- | --- | --- | --- | --- | --- | --- | --- | --- |
| **Rearfoot** | **half-shoe** | 102.9  **(**79.0 129.9) § | **0.021** | 205.0  **(**175.0 257.5) |  | 61.1  **(**47.9 77.9) |  | 37.9 *  **(**29.9 48.3) | **0.044** | 77.6  **(**66.8 87.6) |  | 22.2  **(**17.8 30.4) *§ | **0.007** |
|  | **full-outsole** | 120.3  **(**106.9 134.1) *§ | **0.044** | 245.0  **(**205.0 275.0) |  | 58.1 (50.2 67.4) |  | 36.5 (34.0 41.6) * | **0.026** | 72.9 (67.6 77.4) |  | 18.0 (15.8 21.3) § | **0.045** |
|  | **Control** | 101.1  **(**84.8 116.4) |  | 196.3  **(**175.0 237.5) |  | 51.0 (44.8 59.4) |  | 34.1 (29.0 36.5) |  | 68.8 (58.4 75.2) |  | 15.7 (14.6 19.4) |  |
| **Midfoot** | **half-shoe** | 47.7  **(**41.2 52.8) |  | 67.5  **(**60.0 80.0) |  | 26.7  (23.2 33.1) * | **0.050** | 13.7  **(**11.0 15.7) * | **0.006** | 25.4 (21.4 28.2) *§ | **0.013** | 7.2 (5.8 9.4) *§ | **0.006** |
|  | **full-outsole** | 42.5  **(**32.8 60.0) |  | 68.8  (45.0 82.5) |  | 21.3 (13.8 31.8) |  | 7.0 (5.4 11.3) |  | 12.1 (9.4 18.5) § | **0.018** | 3.4 (2.4 6.2) § | **0.028** |
|  | **Control** | 36.2  **(**30.9 45.4) |  | 57.5 (42.5 65.0) |  | 19.1 (14.9 27.7) |  | 6.1 (4.8 7.5) |  | 11.7 (8.9 13.6) |  | 3.3 (2.2 4.5) |  |
|  | **half-shoe** | 76.9  (60.7 95.5) * | **0.010** | 170.0  (120.0 255.0) * | **0.027** | 52.8  (32.8 58.9) * | **0.009** | 28.4  (23.6 32.1) *§ | **0.001** | 62.1  (52.1 65.4) *§ | **<0.001** | 18.0  (14.7 21.9) *§ | **0.002** |
| **Forefoot** | **full-outsole** | 97.3  (84.5 111.2) |  | 185.0  (152.5 207.5) |  | 63.3  (52.7 68.1) |  | 37.4  (34.5 46.7) § | **0.002** | 86.9  (76.3 92.7) § | **0.019** | 24.9  (20.4 28.4) § | **0.002** |
|  | **Control** | 99.5  (86.6 136.8) |  | 217.5  (197.5 265.0) |  | 63.2  (56.1 82.7) |  | 39.6  (36.7 42.8) |  | 93.5  (88.9 99.7) |  | 24.6  (22.5 27.0) |  |
| **First Metatarsal** | **half-shoe** | 50.4  **(**41.2 72.6) * | **0.001** | 92.5 (70.0 112.5) * | **0.001** | 26.6 (20.0 37.5) * | **0.022** | 6.0 (4.9 7.8) * | **0.001** | 11.3 (9.1 14.7) * | **<0.001** | 2.8 (2.3 4.1) * | **0.012** |
|  | **full-outsole** | 70.9  **(**59.6 84.3) |  | 115.0 (102.5 135.0) |  | 32.8 (27.8 41.6) |  | 8.4 (5.5 10.5) |  | 16.5 (11.2 19.7) |  | 4.1 (2.7 4.8) |  |
|  | **Control** | 89.8  **(**66.9 118.8) |  | 170.0 (122.5 190.0) |  | 43.0 (29.9 54.9) |  | 11.8 (8.1 14.1) |  | 22.1 (15.5 27.2) |  | 4.8 (3.6 6.6) |  |
| **Hallux** | **half-shoe** | 64.7  **(**34.5 91.6) *§ | **0.004** | 116.3 (47.5 255.0) * | **0.042** | 21.7 (4.8 35.3) *§ | **0.001** | 4.7 (1.2 6.2) *§ | **0.006** | 9.6 (1.9 18.2) |  | 1.5 (0.2 2.5) *§ | **0.008** |
|  | **full-outsole** | 97.1  **(**72.9 104.6) § | **0.014** | 178.8 (137.5 207.5) |  | 33.7 (24.4 52.9) § | **0.020** | 6.7 (5.5 7.8) § | **0.008** | 13.4 (9.8 19.3) |  | 2.4 (1.9 3.8) § | **0.013** |
|  | **Control** | 99.3  **(**79.4 118.8) |  | 212.5 (162.5 242.5) |  | 41.1 (29.3 58.2) |  | 7.0 (5.5 8.3) |  | 15.6 (11.3 20.0) |  | 2.8 (2.0 3.7) |  |
| **Total**  **Foot** | **half-shoe** | 131.8  **(**107.8 146.5) |  | 246.3 (195.0 270.0) |  | 93.5 (78.5 108.2) |  | 68.1 (64.0 75.3) |  | 92.8 (87.2 95.5)* | **0.012** | 50.3 (44.1 54.5) |  |
|  | **full-outsole** | 143.8  **(**131.5 172.1) |  | 246.3 (205.0 277.5) |  | 100.5 (83.1 117.3) |  | 69.3 (62.1 73.8) |  | 93.4 (84.8 98.8) |  | 47.5 (42.8 50.7) |  |
|  | **Control** | 146.0  **(**134.9 166.4) |  | 236.3 (207.5 270.0) |  | 97.7 (89.1 119.1) |  | 66.6 (63.9 73.0) |  | 97.0 (93.5 103.0) |  | 45.9 (39.4 47.8) |  |

## Additional file 1: Table S1a. Pedobarographic parameters in the ipsilateral side

Main pedobarographic parameters in different foot regions and in the total foot in the right side. Median (25% 75%) values were calculated across 30 samples for each shoe condition.

* denotes statistically significant difference (p<0.05) between any FOS and control. § denotes statistically significant difference (p<0.05) between the two FOS.
