# Supplementary material for: Pedobarographic and kinematic analysis in the functional evaluation of two post-operative forefoot offloading shoes
Source: J Foot Ankle Res. 2015 Oct 29;8:59. doi: 10.1186/s13047-015-0116-3 (PMC4625618; doi:10.1186/s13047-015-0116-3)
Supplement: Additional file 1: Tables S1. — aPedobarographic parameters in the ipsilateral side. Main pedobarographic parameters in different foot regions and in the total foot in the right side. Median (25 % 75 %) values were calculated across 30 samples for each shoe condition.*denotes statistically significant difference (p<0.05) between any FOS and control. § denotes statistically significant difference (p<0.05) between the two FOS. b Pedobarographic parameters in the contralateral side.Main pedobarographic parameters at different foot regions and in the total foot in the left side (where the control shoe was worn). Median (25 % 75 %) values were calculated across 30 samples for each of the three shoe conditions on the right side. *denotes statistically significant difference (p<0.05) between any FOS and control. (ZIP 41 kb) [file 13047_2015_116_MOESM1_ESM.zip › Additional file 1/1514672762170943_add4.docx]

|  |  | **Mean pressure**  **[kPa]** | **p** | **Peak pressure**  **[kPa]** | **p** | **PTI**  **[kPa*s]** | **p** | **Mean force**  **[% BW]** | **p** | **Maximum force**  **[%BW]** | **p** | **FTI**  **[% BW*s]** | **p** |
| --- | --- | --- | --- | --- | --- | --- | --- | --- | --- | --- | --- | --- | --- |
| **Rearfoot** | **half-shoe** | 102.3 (94.3 120.3) |  | 218.8 (190.0 235.0) |  | 56.7 (47.0 59.4) |  | 32.9 (30.4 41.4) |  | 70.6 (60.0 82.4) |  | 18.1 (14.4 21.1) |  |
|  | **full-outsole** | 96.6 (85.6 107.2) |  | 186.3 (167.5 215.0) |  | 49.2 (44.6 53.7) |  | 32.2 (30.5 34.9) |  | 65.6 (57.0 72.5) |  | 16.0 (14.1 19.5) |  |
|  | **control** | 95.1 (83.5 106.9) |  | 192.5 (175.0 222.5) |  | 49.9 (43.5 57.8) |  | 31.9 (27.5 36.2) |  | 71.2 (67.1 76.3) |  | 17.0 (13.6 20.1) |  |
| **Midfoot** | **half-shoe** | 36.7 (30.7 51.8) |  | 60.0 (52.5 80.0) |  | 20.5 (16.4 28.0) |  | 6.7 (4.9 8.5) |  | 14.4 (9.1 17.8) |  | 3.6 (2.5 5.4) |  |
|  | **full-outsole** | 38.9 (28.8 49.6) |  | 60.0 (42.5 85.0) |  | 21.5 (13.7 30.6) |  | 7.0 (4.7 9.3) |  | 13.7 (11.0 18.0) |  | 3.8 (2.3 5.7) |  |
|  | **control** | 37.7 (25.3 50.4) |  | 61.3 (37.5 77.5) |  | 19.6 (12.9 29.6) |  | 6.7 (4.4 8.5) |  | 12.1 (9.1 16.8) |  | 3.4 (1.8 4.7) |  |
| **Forefoot** | **half-shoe** | 115.1 (89.5 125.3) |  | 247.5 (205.0 292.5) |  | 73.6 (58.8 85.5) |  | 43.6 (40.6 49.5) |  | 97.4 (91.4 105.4) |  | 29.1 (24.3 33.5) |  |
|  | **full-outsole** | 115.6 (92.3 128.3) |  | 247.5 (220.0 267.5) |  | 72.4 (60.1 79.8) |  | 46.1 (40.7 51.6) |  | 99.5 (92.2 109.3) |  | 28.5 (24.6 31.7) |  |
|  | **control** | 105.0 (94.8 121.7) |  | 217.5 (197.5 285.0) |  | 65.4 (55.4 77.2) |  | 43.2 (39.3 48.1) |  | 101.3 (92.4 105.1) |  | 25.7 (23.5 30.2) |  |
| **First Metatarsal** | **half-shoe** | 84.1 (68.6 94.5) |  | 146.3 (110.0 170.0) |  | 39.4 (32.4 49.2) |  | 10.4 (9.4 12.3) |  | 19.0 (17.5 22.9) |  | 4.8 (4.1 6.3) |  |
|  | **full-outsole** | 75.9 (69.5 97.2) |  | 145.0 (112.5 170.0) |  | 37.5 (29.8 49.5) |  | 9.3 (7.0 12.7) |  | 17.8 (14.8 22.8) |  | 5.1 (2.9 5.9) |  |
|  | **control** | 78.0 (61.3 89.5) |  | 126.3 (107.5 170.0) |  | 35.6 (29.3 41.3) |  | 9.9 (6.4 12.6) |  | 19.4 (14.624.3) |  | 4.4 (3.6 5.4) |  |
| **Hallux** | **half-shoe** | 117.0 (86.1 144.6) |  | 232.5 (145.0 292.5) |  | 49.1 (38.2 69.6) |  | 8.2 (6.7 9.9) |  | 17.7 (12.9 20.2) |  | 3.6 (3.0 4.5) |  |
|  | **full-outsole** | 121.0 (81.9 136.9) |  | 227.5 (155.0 262.5) |  | 49.2 (36.2 65.5) |  | 8.4 (6.4 9.3) |  | 16.5 (15.2 21.0) |  | 3.5 (2.4 4.3) |  |
|  | **control** | 114.2 (81.3 128.2) |  | 213.8 (137.5 280.0) |  | 37.6 (25.5 56.8) |  | 8.1 (6.3 10.0) |  | 17.1 (13.4 20.1) |  | 3.2 (2.2 3.8) |  |
| **Total**  **Foot** | **half-shoe** | 150.2 (138.2 165.8) |  | 251.3 (235.0 292.5) |  | 109.2 (98.6 126.7) * |  | 69.9 (64.1 77.8) |  | 100.7 (94.1 109.0) |  | 49.7 * (44.6 59.3) | **0.009** |
|  | **full-outsole** | 149.5 (137.3 159.8) |  | 248.8 (227.5 267.5) |  | 105.5 (94.3 111.4) |  | 71.2 (64.5 74.8) |  | 102.8 (97.6 110.6) |  | 49.6 (45.2 52.3) |  |
|  | **control** | 147.2 (137.8 159.6) |  | 236.3 (212.5 285.0) |  | 100.6 (89.4 106.1) |  | 70.7 (65.3 76.8) |  | 103.4 (98.4 107.6) |  | 47.3 (40.8 53.0) |  |

## Additional file 1: Table S1b. Pedobarographic parameters in the contralateral side

Main pedobarographic parameters at different foot regions and in the total foot in the left side (where the control shoe was worn). Median (25% 75%) values were calculated across 30 samples for each of the three shoe conditions on the right side.

* denotes statistically significant difference (p<0.05) between any FOS and control. § denotes statistically significant difference (p<0.05) between the two FOS.
