# Supplementary figures and images for: Pedobarographic and kinematic analysis in the functional evaluation of two post-operative forefoot offloading shoes
Source: J Foot Ankle Res. 2015 Oct 29;8:59. doi: 10.1186/s13047-015-0116-3 (PMC4625618; doi:10.1186/s13047-015-0116-3)

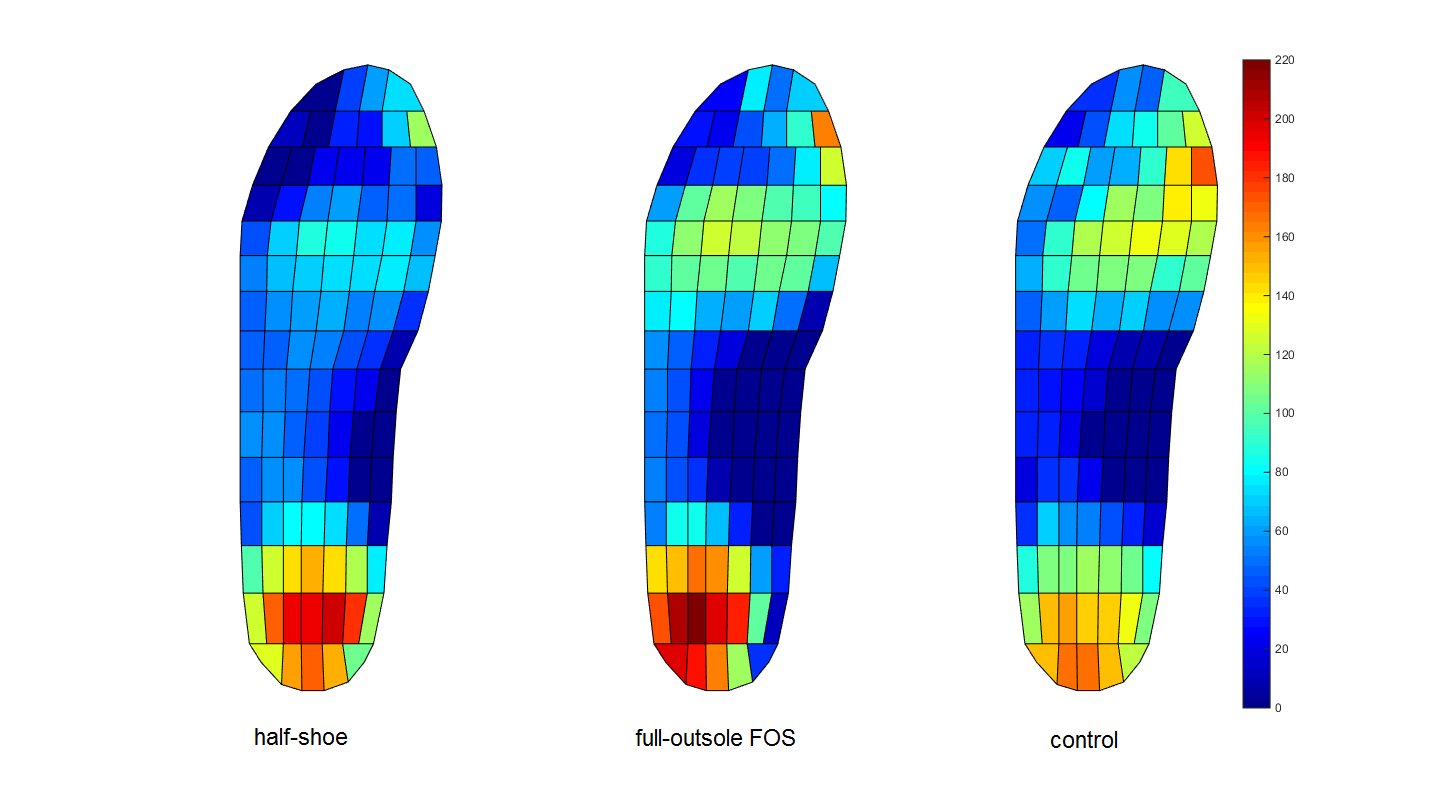

Supplement: Additional file 2: Figure S1. — Sensor-based peak pressure. For each shoe condition, color-map of the median of the peak pressure (kPa) for each sensor across all steps and all participants. (PNG 66 kb) [file 13047_2015_116_MOESM2_ESM.png]
