# Supplementary material for: Pedobarographic and kinematic analysis in the functional evaluation of two post-operative forefoot offloading shoes
Source: J Foot Ankle Res. 2015 Oct 29;8:59. doi: 10.1186/s13047-015-0116-3 (PMC4625618; doi:10.1186/s13047-015-0116-3)
Supplement: Additional file 3: Table S2. — Kinematic parameters in the contralateral limb.Range of motion and maximum/minimum sagittal-plane rotation angles [deg] at the hip, knee and ankle joint in the left side (where the control shoe was worn), for each of the three shoe conditions on the right side. Kinematic parameters were determined according to [18]. (DOCX 14 kb) [file 13047_2015_116_MOESM3_ESM.docx]

|  |  | **Half-Shoe**  [deg] | **Full-Outsole**  [deg] | **Control**  [deg] | **p**  **HS vs FO** | **p**  **HS vs CON** | **p**  **FO vs CON** |
| --- | --- | --- | --- | --- | --- | --- | --- |
| **HIP** | ROM  sagittal-plane | 46.1  (44.1 48.0) | 46.4  (43.7 48.3) | 46.3  (43.3 48.6) | 0.883 | 0.629 | 0.898 |
|  | ROM  frontal-plane | 13.2  (11.7 14.2) | 12.8  (11.3 13.8) | 13.1  (11.4 13.9) | 0.981 | 0.968 | 0.998 |
|  | ROM  transverse-plane | 8.7  (7.0 12.3) | 9.3  (7.4 13.5) | 9.2  (7.8 13.3) | 0.510 | 0.415 | 0.986 |
|  | Max flexion in swing | 31.3  (29.1 35.0) | 32.7  (29.2 35.5) | 32.8  (30.7 34.5) | 0.975 | 0.556 | 0.692 |
|  | Max extension in stance | -13.5  (-17.9 -9.5) | -12.4  (-16.9 -10.0) | -14.0  (-17.3 -9.5) | 0.602 | 0.928 | 0.825 |
|  |  |  |  |  |  |  |  |
| **KNEE** | ROM  sagittal-plane | 70.4  (66.0 73.6) | 70.9  (67.5 73.6) | 70.7  (68.9 74.6) | 0.702 | 0.231 | 0.682 |
|  | ROM  frontal-plane | 7.7  (6.1 8.8) | 8.1  (5.8 9.3) | 9.8  (7.1 11.3) | 0.939 | 0.596 | 0.391 |
|  | ROM  transverse-plane | 16.1  (13.8 18.0) | 17.9  (13.2 21.2) | 16.9  (13.8 21.5) | 0.589 | 0.397 | 0.946 |
|  | Max flexion at loading response | 14.8  (6.9 17.7) | 10.5  (5.6 16.4) | 11.6  (6.2 16.4) | 0.833 | 0.886 | 0.994 |
|  | Max extension in stance | 2.2  (-0.6 5.1) | 2.1  (-0.2 4.2) | 1.4  (-1.5 4.3) | 0.985 | 0.692 | 0.790 |
|  |  |  |  |  |  |  |  |
| **ANKLE** | ROM  sagittal-plane | 28.6  (26.7 31.3) | 29.8  (27.6 35.1) | 28.5  (26.3 34.2) | 0.546 | 0.939 | 0.348 |
|  | ROM  frontal-plane | 8.4  (7.1 10.6) | 9.2  (7.3 11.8) | 9.0  (7.3 12.7) | 0.987 | 0.922 | 0.971 |
|  | ROM  transverse-plane | 14.6  (12.9 18.1) | 14.8  (13.1 17.8) | 13.2  (11.1 16.6) | 0.990 | 0.292 | 0.231 |
|  | Max dorsiflexion in stance | 14.6  (7.7 17.5) | 13.4  (8.1 16.8) | 11.6  (7.8 19.2) | 0.503 | 0.959 | 0.676 |
|  | Max plantarflexion at loading response | -3.9  (-8.8 -1.7) | -5.1  (-8.3 -2.5) | -5.2  (-9.3 -3.8) | 0.081 | 0.183 | 0.922 |

**Additional file 3: Table S2 Supplementary. Kinematic parameters in the contralateral limb.**

Range of motion and maximum/minimum sagittal-plane rotation angles [deg] at the hip, knee and ankle joint in the left side (where the control shoe was worn), for each of the three shoe conditions on the right side. Kinematic parameters were determined according to [18].

* denotes statistically significant difference between any FOS and control (p<0.05). § denotes statistically significant difference between the two FOS (p<0.05).
